# The impact of maternal health and lifestyle on low birth weight: a prospective cohort study

**DOI:** 10.1186/s13052-025-02080-x

**Published:** 2025-07-10

**Authors:** Xiaorui Ruan, Kebin Chen, Ziye Li, Jianhui Wei, Ye Chen, Qi Zou, Yuan Peng, Manjun Luo, Mengting Sun, Tingting Wang, Jiabi Qin

**Affiliations:** 1 Department of Epidemiology and Health Statistics, Xiangya School of Public Health, Central South University, Changsha, 410013 China; 2 Department of Epidemiology and Health Statistics, School of Public Health, Kunming Medical University, Kunming, 650500 China

**Keywords:** Low birth weight, Associated factor, Gestational complication, Gestational nutrition, Lifestyle behavior, Population attributable fraction, Preterm birth

## Abstract

**Background:**

To explore maternal pregestational and periconceptional factors associated with low birth weight in offspring and inform the development of targeted interventions.

**Methods:**

A prospective birth cohort involving 34,104 pregnant women and their offspring was constructed. The participants were enrolled during 8–14 gestational weeks and followed up at 3 months postpartum. Modified Poisson regression with robust error variances was employed to examine the associations between low birth weight and various maternal factors, including demographics, medical history, obstetric factors, lifestyle behaviors, nutrition, and environmental exposures.

**Results:**

The incidence of low birth weight was 8.9% (95%CI: 8.6–9.2). Maternal demographic factors, including advanced gestational age (≥ 35 years, *RR* = 1.14), urban residence (*RR* = 1.74) and a lower education level, were found to be associated with low birth weight. Pregestational medical and behavioral factors significantly increased the risk of low birth weight, including multiparity, a history of preterm birth, diseases such as tuberculosis and syphilis, and alcohol consumption (*RR*s: 1.71, 1.56, 2.27, 2.25, and 1.54, respectively). Additionally, periconceptional factors also significantly contributed to low birth weight, including medical factors (infections, gestational complications such as preeclampsia, a lack of folic acid supplementation; *RR*s: 2.36, 5.97, 1.48), nutritional factors (being underweight before conception, weight gain < 10 kg during pregnancy, imbalanced diet; *RR*s: 1.59, 2.42, 1.34), behavioral factors (alcohol consumption and moderate-to-high physical activity; *RR*s: 1.23 and 1.22), and exposure to renovation pollutants (*RR* = 1.21). Overall, observed modifiable risk factors accounted for 40.92% of low birth weight cases, with a greater proportion found in mothers with advanced gestational age than in those under 35 years (44.61% vs. 31.91%). Among these factors, achieving adequate weight gain during pregnancy (≥ 10 kg) could prevent 20.59% (18.68–22.45) of cases. Furthermore, the incidence of low birth weight may be effectively reduced through maintaining a balanced diet, supplementing folic acid, and avoiding excessive physical activity during pregnancy.

**Conclusions:**

Mothers at risk for delivering low-birth-weight infants can be identified based on pregestational and periconceptional factors. This could be prevented through targeted interventions, including nutritional and behavioral measures. Tailored interventions should be prioritized by antenatal care providers.

**Trial registration:**

The study was retrospectively registered in Chinese Clinical Trial Registry Center on 06/14/2018, registration number: ChiCTR1800016635, available at: https://www.chictr.org.cn/showproj.html?proj=28300.

**Supplementary Information:**

The online version contains supplementary material available at 10.1186/s13052-025-02080-x.

## Significance

**What is already known on this topic**

Low birth weight is a critical problem that affects children’s long-term health and is reportedly associated with maternal demographics, nutrition, and gestational complications.

**What this study adds**

1. This study expands the knowledge regarding the associations between maternal pregestational factors and low birth weight, such as history of various diseases and preterm birth. Additionally, several periconceptional factors significantly associated with low birth weight were identified, including periconceptional nutritional factors, a lack of folic acid supplementation, gestational complications, infections and unhealthy lifestyle behaviors during the first trimester.
2. The modifiable risk factors identified in this study accounted for 40.92% of low-birth-weight cases. Notably, achieving adequate weight gain during pregnancy (≥ 10 kg) had the potential to prevent 20.59% (18.68–22.45) of cases.

**How this study might affect research, practice or policy**

This research provides evidence for targeted interventions for low birth weight. Low birth weight can be prevented through nutritional and lifestyle interventions during pregnancy, such as achieving adequate weight gain, maintaining a balanced diet, abstaining from tobacco and alcohol consumption, and avoiding moderate-to-high physical activity.

## Background

Low birth weight refers to newborns with weight < 2500 g at birth independent of gestational week and serves as a common measure of neonatal morbidity, mortality, and pregnancy outcomes [1–3]. The global prevalence of low birth weight is estimated to be 14.6% (20.5 million cases), with the highest estimates observed in Southern Asia (26.4%) and the lowest in North America (7%) and Europe (4–7%) [4, 5]. In China, the overall prevalence of low birth weight is 5.15%, with regional variations ranging from 3.75–7.34% [6]. Despite the improved survival of low birth weight infants, recent publications have warned of their later life consequences, including cardiovascular risk [7], metabolic disorders [8], renal diseases [9], and cognitive impairment [10]. Therefore, low birth weight remains a critical public health issue, and investigations into associated factors and the development of interventions accordingly are needed.

Studies have been conducted to explore associated factors prior to or during gestation. A lower socioeconomic status, advanced gestational age, poor education, and living in rural areas were found to be associated with low birth weight [11–14]. Accumulating evidence has shown that maternal nutritional status, indicated by BMI before/during gestation and anemia, affects birth weight [14–17]. Moreover, gestational complications such as gestational hypertension, anemia and thyroid disorders have been reported to be predictors of low birth weight [18, 19]. Overall, half of low birth weight cases were attributed to modifiable factors, with smoking during pregnancy reported to be the most prominent determinant [20]. However, the effects of pregestational diseases, a history of adverse birth outcomes, periconceptional medication, and environmental exposures on birth weight have not yet been reported. In addition, the specific contribution of each factor, particularly modifiable factors with preventive value, remains unclear.

Hence, the aims of this prospective cohort study were as follows: 1) to estimate the incidence of low birth weight in China; 2) to explore a broad range of potential factors, especially modifiable factors, for low birth weight; and 3) to evaluate the burden of modifiable determinants on low birth weight, offering suggestions for targeted interventions.

## Methods

This study follows the reporting guidelines of the Strengthening the Reporting of Observational Studies in Epidemiology (STROBE). The current project was performed in line with the principles of the Declaration of Helsinki and approved by the Ethics Committee of Xiangya School of Public Health, Central South University (grant number: XYGW-2018–36). Written informed consent was obtained from each participant. Furthermore, it has been registered in the Chinese Clinical Trial Registry Center (registration number: ChiCTR1800016635).

### Study design

This was a prospective cohort study of pregnant women (≥ 18 years) who underwent their first prenatal care between 8 and 14 weeks of gestation at the Hunan Provincial Maternal and Child Health Care Hospital, between March 13, 2013 and December 31, 2019. Gestational weeks were calculated on the basis of previous menstrual period data or determined via ultrasonography regarding irregular menstruation. Notably, the invited pregnant women all intended to continue receiving antenatal care throughout their pregnancy. Upon enrollment, trained investigators conducted in-person interviews with participants who provided informed consent using a standardized questionnaire. To control for recall bias, medical records, as well as the perinatal health care handbook (PHCH), provide confirmation of the disease diagnosis [21].

**Fig. 1:**
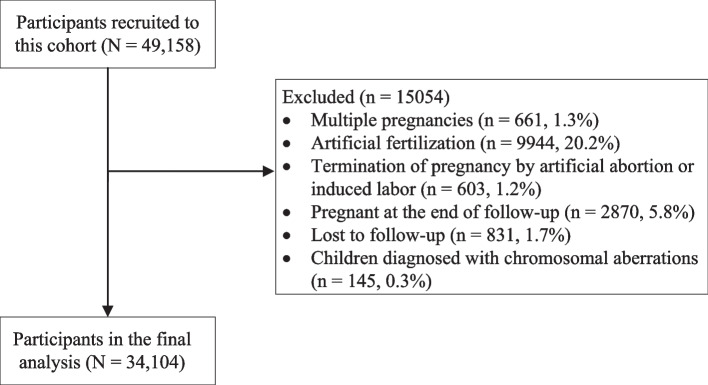
Flow chart of this birth cohort between March 13, 2013 and December 31, 2019

Each subject was followed up from recruitment until 3 months postpartum, and an investigation was performed face-to-face during each prenatal examination. Additionally, information of gestational weeks, complications, and neonatal outcomes was retrieved from the electronic medical records system. A total of 831 participants lost to follow-up, mainly due to changes in residence, were dismissed from our dataset. We further excluded participants with multiple pregnancies (*n* = 661, 1.3%), artificial fertilization (9944, 20.2%), termination of pregnancy by artificial abortion or induced labor (603, 1.2%), who were still pregnant at the end of follow-up (2870, 5.8%), and whose children were diagnosed with chromosomal aberrations (145, 0.3%). In total, 34,104 participants were included in the analysis (Fig. 1).

### Outcome of interest

According to the World Health Organization, low birth weight was diagnosed as a birth weight < 2500 g measured within the first hour post-birth, irrespective of gestational age (available at https://www.who.int/publications/i/item/WHO-NMH-NHD-14.5). We additionally divided low birth weight into very low birth weight (VLBW, < 1500 g) and extremely low birth weight (ELBW, < 1000 g).

### Exposures of interest

A self-designed composite questionnaire was used to assess demographic factors, history of previous pregnancy, pregestational diseases, diseases during the first trimester, gestational complications, periconceptional medication, periconceptional nutrition, lifestyle behaviors, and periconceptional exposure to environmental hazards. These factors were categorized into two aspects, pregestational exposures and periconceptional exposures. The periconceptional period was defined as 3 months before conception and during pregnancy.

Maternal height and weight before conception were recorded to calculate body mass index (BMI). A value ranging 18.5–24.0 kg/m^2^ was considered as healthy for reference, < 18.5 kg/m^2^ as underweight, 24.0–28.0 kg/m^2^ as overweight, and > 28.0 kg/m^2^ as obese, based on Chinese adult criteria [22]. Weight gain during pregnancy was measured by the difference between weight at the last and first antenatal visits using a calibrated electronic scale, with the mother wearing light clothing but no shoes at all the time of measurement. Folic acid supplementation was defined as taking at least 0.4 mg of folic acid daily for more than 5 days per week continuously before conception and throughout the first trimester of pregnancy [23]. The participants were classified into balanced and imbalanced diet groups based on the core recommendations of the Chinese Dietary Guidelines and their self-reported intakes of the three major macronutrients (carbohydrates, protein, and fat) during gestation [24]. Regarding lifestyle behaviors, smoking was defined as the average number of cigarettes actively smoked ≥ 1 per day, and passive smoking as exposure to smoke for ≥ 15 min per day and for more than one day per week (either at home or in the workplace) [25].The definition of drinking was consuming any kind of alcohol (≥ 2 g of alcohol) more than once per day [26]. According to the Pregnancy Physical Activity Questionnaire-Chinese version (PPAQ-C), metabolic equivalents (MET-min/week) were used to assess the total amount of physical activity involved in each item of the questionnaire. A score of ≥ 600 METs-min/week was classified as moderate-to-high physical activity, and < 600METs-min/week was considered low physical activity [27].

### Statistical analysis

Continuous and categorical variables are presented as medians and standard deviations and as cases and proportions, respectively. To explore potential risk factors for low birth weight, modified Poisson regression with robust error variances was conducted to assess the association between each exposure of interest and low birth weight due to the violated rare disease assumption in our binary outcome. Statistically significant factors were subsequently analyzed in multivariable models through modified Poisson regression, and relative risks (*RRs*) with corresponding 95% confidence intervals (*CIs*) were estimated. The Cochran-Armitage test was used to examine trends. Collinearity diagnosis consisted of the *K-M–O* statistic and the Bartlett test was performed within each dimension to determine multicollinearity between categorical variables. Since the majority of low-birth-weight cases stem from preterm birth, we conducted subgroup analysis on preterm births (defined as those born before 37 weeks of gestation) and non-preterm infants.

Furthermore, we computed the population attributable fractions (*PAFs*) to determine the percentage of low birth weight that could have been prevented if undesired modifiable variables were eliminated, presuming that the observed associations were causal. *PAFs* were obtained for potential modifiable risk factors identified in modified Poisson regression models, with corresponding 95%*CIs* generated from Monte Carlo simulation [28]. Statistical analyses were performed in R 4.3.3, and two-tailed *P* < 0.05 indicated a statistically significant difference.

## Results

Among the total of 34,104 eligible births, the mean birth weight was 3143.8 ± 554.1 g, and male neonates had higher birth weights than their female counterparts (3190.9 ± 557.3 vs. 3092.8 ± 544.1, *P* < 0.001). The incidence of low birth weight, VLBW, and ELBW in the offspring was 8.9% (95%*CI*: 8.6–9.2, *n* = 3020), 1.5% (1.4–1.6, *n* = 505), and 0.4% (0.3–0.5, *n* = 140), respectively. Besides, 11.8% of the infants (11.5–12.2, *n* = 4041) were classified as preterm births based on their gestational age.

### Univariate analysis

Univariate modified Poisson regression analysis revealed pregestational and periconceptional factors associated with low birth weight, as presented in Table 1 and Table 2. The results of the collinearity analysis did not indicate the presence of multicollinearity, with all *K-M–O* statistics being less than 0.6. Therefore, subsequent measures to reduce dimensionality were unnecessary, as detailed in Supplementary Table 1 [see Additional file 1].

**Table 1:** Pregestational factors of low birth weight and their univariate associations conducted by modified Poisson regression with robust error variances

Dimension	Variables	Number in cohort	Number in LBW group	*RR* (95%*CI*)	*P*
**Demographic factors**					
	Age at pregnancy				
	< 35 years	26,445	2141	1.00 (reference)	
	≥ 35 years	7659	879	1.42 (1.32–1.53)	<.001
	Ethics				
	Minorities	448	44	1.00 (reference)	
	Han	33,656	2976	0.90 (0.68–1.19)	0.467
	Education degree				
	Primary or middle school	2547	588	1.00 (reference)	
	Senior high or technical school	9695	1030	0.46 (0.42–0.50)	<.001
	Junior college	15,594	920	0.26 (0.23–0.28)	<.001
	Bachelor’s degree and above	6268	482	0.33 (0.30–0.37)	<.001
	Residence				
	Rural area	13,030	1642	1.00 (reference)	
	Urban area	21,074	1378	1.93 (1.80–2.06)	<.001
**History of previous pregnancy**					
	Parity				
	1 (primipara)	10,493	687	1.00 (reference)	
	2–5	22,179	2084	1.44 (1.32–1.56)	<.001
	≥ 6	1432	249	2.66 (2.32–3.04)	<.001
	History of gestational complication	6654	602	1.03 (0.94–1.12)	0.539
	History of stillbirth	466	48	1.17 (0.89–1.53)	0.266
	History of preterm birth	302	78	2.97 (2.44–3.60)	<.001
	History of low birth weight	344	42	1.38 (1.04–1.84)	0.026
	History of neonatal death	362	44	1.38 (1.04–1.82)	0.024
**Pregestational diseases**					
	Hypertension	280	106	4.39 (3.77–5.13)	<.001
	Diabetes mellitus	194	20	1.17 (0.77–1.77)	0.472
	Heart disease	497	71	1.63 (1.31–2.02)	<.001
	Kidney disease	323	59	2.08 (1.65–2.63)	<.001
	Liver disease	229	19	0.94 (0.61–1.44)	0.766
	Carcinoma	88	10	1.28 (0.72–2.30)	0.402
	Hematological disease	742	117	1.81 (1.53–2.15)	<.001
	Systemic lupus erythematosus, SLE	340	57	1.91 (1.50–2.43)	<.001
	Antiphospholipid syndrome, APL	70	13	2.10 (1.29–3.44)	0.003
	Hyperlipidemia	32	0	NA	
	Thyroid disease	1098	61	0.62 (0.48–0.79)	<.001
	Hepatitis B, HBV	2084	185	1.00 (0.87–1.16)	0.971
	Tuberculosis	184	33	2.04 (1.49–2.78)	<.001
	Syphilis	107	28	2.97 (2.16–4.10)	<.001
	Acquired immune deficiency syndrome, AIDS	6	0	NA	
**Pregestational lifestyle**					
	Smoking	342	44	1.46 (1.11–1.93)	0.008
	Passive smoking	4938	468	1.08 (0.99–1.99)	0.095
	Drinking	580	110	2.18 (1.84–2.59)	<.001

*CI* Confidence interval, *LBW* Low birth weight, *RR* Relative risk

**Table 2:** Periconceptional factors of low birth weight and their univariate associations conducted by modified Poisson regression with robust error variances

Dimension	Variables	Number in cohort	Number in LBW group	*RR* (95%*CI*)	*P*
**Diseases during the first trimester**					
	Have a cold	3136	297	1.08 (0.96–1.21)	0.202
	Fever	1203	108	1.01 (0.84–1.22)	0.879
	Systematic infection	51	13	2.89 (1.80–4.62)	<.001
	Respiratory infection	386	55	1.62 (1.27–2.07)	<.001
	Urinary tract infection	99	27	3.10 (2.24–4.28)	<.001
	Reproductive tract infection	757	88	1.32 (1.08–1.61)	0.006
**Gestational complications**					
	Hyperemesis gravidarum	47	5	1.20 (0.52–2.75)	0.664
	Gestational anemia	2436	256	1.20 (1.07–1.36)	0.003
	Preeclampsia	788	488	8.15 (7.63–8.71)	<.001
	Gestational diabetes mellitus	5430	495	1.04 (0.94–1.13)	0.460
	Hyperlipidemia	375	34	1.02 (0.74–1.41)	0.885
	Gestational hyperthyroidism	636	32	0.56 (0.40–0.79)	<.001
	Gestational hypothyroidism	4787	343	0.78 (0.70–0.87)	<.001
	Intrahepatic cholestasis of pregnancy	669	117	2.01 (1.70–2.38)	<.001
	Venous thromboembolism of pregnancy	72	14	2.20 (1.37–3.53)	0.001
	Placenta previa	606	34	0.63 (0.45–0.87)	0.006
	Placental abruption	144	46	3.65 (2.87–4.64)	<.001
	Premature rupture of membrane	1094	144	1.51 (1.29–1.77)	<.001
**Periconceptional medication**					
	Lack of folic acid supplementation	1548	242	1.83 (1.62–2.07)	<.001
	Oral contraceptives intake 3 months before conception	630	40	0.71 (0.53–0.96)	0.028
	Ovulation stimulants intake 3 months before conception	1516	164	1.23 (1.06–1.43)	0.006
	Macrolides antibiotics intake 3 months before conception	998	108	1.23 (1.03–1.48)	0.025
	Antidepressants intake 3 months before conception	964	96	1.13 (0.93–1.37)	0.219
	Macrolides antibiotics intake during the first trimester	828	80	1.09 (0.89–1.35)	0.406
	Antidepressants intake during the first trimester	824	76	1.04 (0.84–1.30)	0.706
**Periconceptional nutrition**					
	BMI before conception				
	< 18.5 kg/m^2^	4920	551	1.41 (1.29–1.54)	<.001
	18.5–23.9 kg/m^2^	23,751	1890	1.00 (reference)	
	24.0–28.0 kg/m^2^	4294	465	1.36 (1.24–1.50)	<.001
	> 28.0 kg/m^2^	925	95	1.29 (1.06–1.57)	0.011
	Weight gain during pregnancy				
	< 10 kg	5645	1055	2.74 (2.55–2.94)	<.001
	10–20 kg	24,672	1683	1.00 (reference)	
	> 20 kg	2787	282	1.09 (0.97–1.23)	0.157
	Imbalanced diet	3898	464	1.41 (1.28–1.54)	<.001
**Periconceptional lifestyle**					
	Smoking during the first trimester	454	50	1.25 (0.96–1.62)	0.100
	Passive smoking during the first trimester	2352	214	1.03 (0.90–1.18)	0.666
	Drinking during the first trimester	498	74	1.70 (1.37–2.10)	<.001
	Moderate-to-high physical activity during the first trimester	6530	668	1.20 (1.11–1.30)	<.001
**Periconceptional exposure to environmental hazards**					
	Exposure to radiation in the workplace	1124	100	1.00 (0.83–1.22)	0.960
	Exposure to environmental hazardous substances near home	616	58	1.06 (0.83–1.36)	0.620
	Renovation at home	1594	178	1.28 (1.11–1.47)	<.001

*BMI* body mass index, *CI* confidence interval, *LBW* low birth weight, *RR*: relative risk

### Multivariate analysis

Multivariate analyses revealed pregestational and periconceptional risk factors associated with low birth weight, as shown in Table 3 and Table 4. Among the demographic factors, advanced gestational age (≥ 35 years, *RR* = 1.14, 95%*CI*: 1.05–1.23) and living in urban areas (*RR* = 1.74, 1.63–1.86) were associated with an elevated risk of low birth weight. Conversely, a higher degree of maternal education (senior high or technical school: *RR* = 0.58; junior college: *RR* = 0.38; bachelor’s degree and above: *RR* = 0.49) was linked to reduced susceptibility to low birth weight in offspring. Multiparity (2–5: *RR* = 1.17; ≥ 6: *RR* = 1.71) was identified as a risk factor for low birth weight. Associations between low birth weight and pregestational diseases were also detected, including hypertension (*RR* = 1.67, 1.39–2.02), kidney diseases (*RR* = 1.76, 1.41–2.20), hematological diseases (*RR* = 1.94, 1.65–2.28), antiphospholipid syndrome (*RR* = 2.11, 1.28–3.47), tuberculosis (*RR* = 2.27, 1.70–3.05), and syphilis (*RR* = 2.25, 1.69–3.01). Moreover, unhealthy lifestyle behaviors such as alcohol consumption were associated with low birth weight (*RR* = 1.54, 1.29–1.84).

**Table 3:** Pregestational factors of low birth weight remained in multivariate modified Poisson regression with robust variance estimator

Dimension	Variables	*RR* (95%*CI*)	*P*
**Demographic factors**			
	Age at pregnancy		
	< 35 years	1.00 (reference)	
	≥ 35 years	1.14 (1.05–1.23)	0.001
	Education degree		
	Primary or middle school	1.00 (reference)	
	Senior high or technical school	0.58 (0.52–0.63)	<.001
	Junior college	0.38 (0.34–0.42)	<.001
	Bachelor’s degree and above	0.49 (0.44–0.55)	<.001
	Residence		
	Rural area	1.00 (reference)	
	Urban area	1.74 (1.63–1.86)	<.001
**History of previous pregnancy**			
	Parity		
	1 (primipara)	1.00 (reference)	
	2–5	1.17 (1.07–1.27)	<.001
	≥ 6	1.71 (1.48–1.96)	<.001
	History of preterm birth	1.56 (1.27–1.90)	<.001
	History of low birth weight	1.21 (0.92–1.60)	0.177
	History of neonatal death	1.23 (0.95–1.59)	0.113
**Pregestational diseases**			
	Hypertension	1.67 (1.39–2.02)	<.001
	Heart disease	1.13 (0.89–1.42)	0.307
	Kidney disease	1.76 (1.41–2.20)	<.001
	Hematological disease	1.94 (1.65–2.28)	<.001
	Systemic lupus erythematosus, SLE	0.98 (0.75–1.28)	0.888
	Antiphospholipid syndrome, APL	2.11 (1.28–3.47)	0.003
	Thyroid disease	0.84 (0.67–1.05)	0.131
	Tuberculosis	2.27 (1.70–3.05)	<.001
	Syphilis	2.25 (1.69–3.01)	<.001
**Pregestational lifestyle**			
	Smoking	1.19 (0.90–1.58)	0.225
	Drinking	1.54 (1.29–1.84)	<.001

*CI* Confidence interval, *RR* Relative risk

**Table 4:** Periconceptional factors of low birth weight remained in multivariate modified Poisson regression with robust variance estimator

Dimension	Variables	*RR* (95%*CI*)	*P*
**Diseases during the first trimester**			
	Systematic infection^*^	2.36 (1.21–4.58)	0.012
	Respiratory infection	1.10 (0.88–1.39)	0.407
	Urinary tract infection	2.54 (1.84–3.49)	<.001
	Reproductive tract infection	1.46 (1.21–1.76)	<.001
**Gestational complications**			
	Gestational anemia	1.14 (1.01–1.28)	0.003
	Preeclampsia	5.97 (5.44–6.56)	<.001
	Gestational hyperthyroidism	0.62 (0.45–0.84)	0.003
	Gestational hypothyroidism	0.85 (0.76–0.95)	0.003
	Intrahepatic cholestasis of pregnancy	1.70 (1.42–2.02)	<.001
	Venous thromboembolism of pregnancy	1.83 (1.23–2.72)	0.003
	Placenta previa	0.53 (0.39–0.72)	<.001
	Placental abruption	2.26 (1.71–2.97)	<.001
	Premature rupture of membrane	1.56 (1.35–1.82)	<.001
**Periconceptional medication**			
	Lack of folic acid supplementation	1.48 (1.32–1.66)	<.001
	Oral contraceptives intake 3 months before conception	0.83 (0.62–1.11)	0.211
	Ovulation stimulants intake 3 months before conception	1.11 (0.97–1.28)	0.128
	Macrolides antibiotics intake 3 months before conception	1.11 (0.93–1.32)	0.247
**Periconceptional nutrition**			
	BMI before conception		
	< 18.5 kg/m^2^	1.59 (1.46–1.74)	<.001
	18.5–23.9 kg/m^2^	1.00 (reference)	
	24.0–28.0 kg/m^2^	0.87 (0.79–0.95)	0.003
	> 28.0 kg/m^2^	0.51 (0.42–0.61)	<.001
	Weight gain during pregnancy		
	< 10 kg	2.42 (2.25–2.60)	<.001
	10–20 kg	1.00 (reference)	
	> 20 kg	0.84 (0.76–0.94)	0.003
	Imbalanced diet	1.34 (1.23–1.47)	<.001
**Periconceptional lifestyle**			
	Drinking during the first trimester	1.23 (1.01–1.49)	0.044
	Moderate-to-high physical activity during the first trimester	1.22 (1.13–1.32)	<.001
**Periconceptional exposure to environmental hazards**			
	Renovation at home	1.21 (1.06–1.38)	0.005

*BMI* body mass index, *CI* confidence interval, *RR* relative risk

^*^ Systemic infection refers to diagnosis such as bacteremia, toxemia, septicemia, and septicopyemia

Maternal medical factors during the periconceptional period were found to be associated with low birth weight in offspring, encompassing infection during the first trimester (systematic infection: *RR* = 2.36; urinary tract infection: *RR* = 2.54; reproductive tract infection: *RR* = 1.46) and a lack of folic acid supplementation (*RR* = 1.48, 1.32–1.66). Significant associations were also observed between gestational complications and an increased risk of low birth weight, including gestational anemia (*RR* = 1.14, 1.01–1.28), preeclampsia (*RR* = 5.97, 5.44–6.56), intrahepatic cholestasis of pregnancy (*RR* = 1.70, 1.42–2.02), venous thromboembolism of pregnancy (*RR* = 1.83, 1.23–2.72), placenta abruption (*RR* = 2.26, 1.71–2.97), and premature rupture of membrane (*RR* = 1.56, 1.35–1.82). In contrast, gestational hyperthyroidism (*RR* = 0.62, 0.45–0.84), gestational hypothyroidism (*RR* = 0.85, 0.76–0.95), and placenta previa (*RR* = 0.53, 0.39–0.72) were associated with a decreased risk of low birth weight.

In addition, maternal periconceptional nutrition was found to be linked to low birth weight. Underweight before conception (< 18.5 kg/m^2^, *RR* = 1.59, 1.46–1.74), insufficient weight gain (< 10 kg, *RR* = 2.42, 2.25–2.60) and an imbalanced diet (*RR* = 1.34, 1.23–1.47) during pregnancy were significantly associated with a higher risk of delivering low birth weight infants. Furthermore, lifestyle behaviors during the first trimester including alcohol consumption (*RR* = 1.23, 1.01–1.49) and moderate-to-high levels of physical activity (*RR* = 1.22, 1.13–1.32) were associated with an increased risk of low birth weight. Of note, a significant association was identified between low birth weight and renovation at home during the periconceptional period (*RR* = 1.21, 1.06–1.38).

### Subgroup analysis

**Fig. 2:**
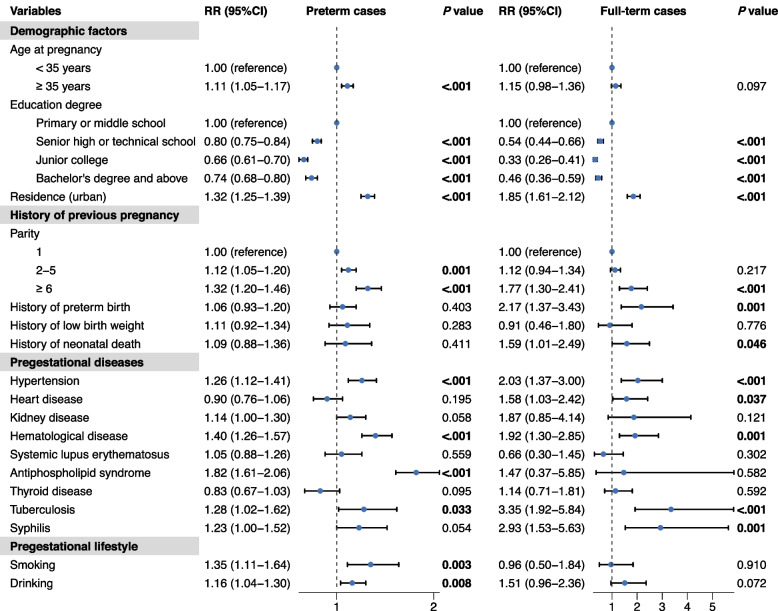
Pregestational factors of low birth weight among preterm and non-preterm infants using multivariate modified Poisson regression with robust variance estimator

**Fig. 3:**
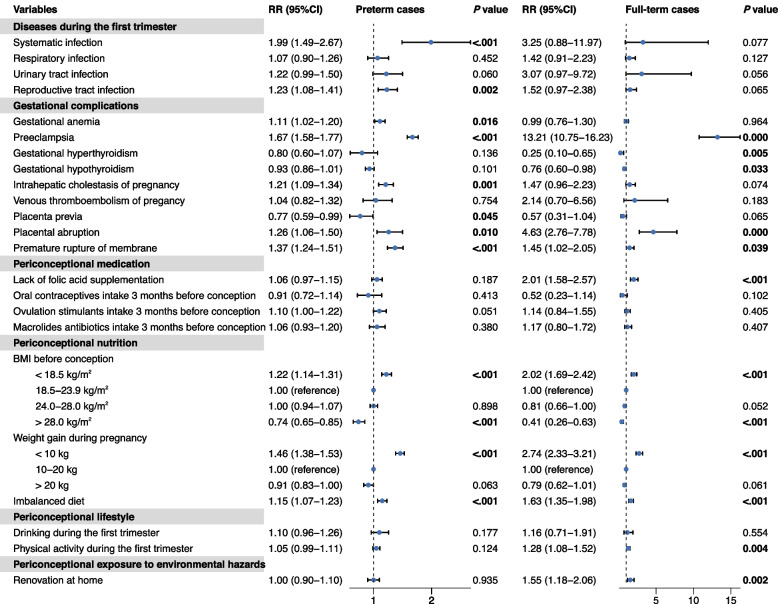
Periconceptional factors of low birth weight among preterm and non-preterm infants using multivariate modified Poisson regression with robust variance estimator

Given the correlation between gestational week and birth weight, subgroup analysis was performed among preterm and non-preterm neonates and yielded different results, as illustrated in Figs. 2 and 3. Regardless of gestational week, a lower maternal education level, urban residence, multiparity, history of pregestational diseases (including hypertension, hematological diseases, and tuberculosis), gestational complications (including preeclampsia, placental abruption, and premature rupture of membrane), being underweight before conception, gaining weight < 10 kg and an imbalanced diet during pregnancy were found to be associated with low birth weight. Notably, the strongest association was observed between preeclampsia and low birth weight in non-preterm neonates (*RR* = 13.21). In addition, factors encompassing advanced gestational age (*RR* = 1.11), pregestational antiphospholipid syndrome (*RR* = 1.82), smoking (*RR* = 1.35) and drinking (*RR* = 1.16) before gestation, systematic (*RR* = 1.99) and reproductive tract infection during the first trimester (*RR* = 1.23), gestational anemia (*RR* = 1.11), and intrahepatic cholestasis of pregnancy (*RR* = 1.21) were significantly associated with an elevated risk of low birth weight among preterm cases. With respect to full-term births, significant associations were found between a greater risk of low birth weight and a history of preterm birth (*RR* = 2.17), a history of neonatal death (*RR* = 1.59), pregestational heart disease (*RR* = 1.58), pregestational syphilis (*RR* = 2.93), a lack of periconceptional folic acid supplementation (*RR* = 2.01), moderate-to-high physical activity during the first trimester (*RR* = 1.28), and renovation at home (*RR* = 1.55).

### PAF estimation for modifiable risk factors

**Fig. 4:**
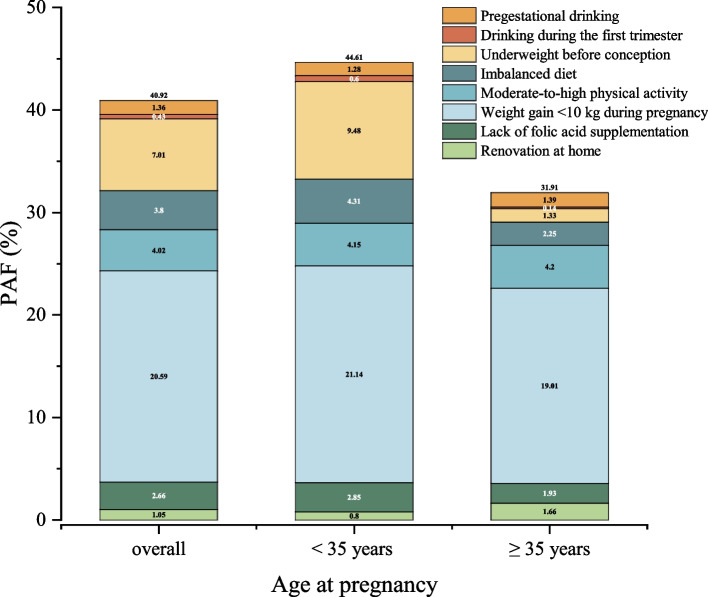
PAF estimation of modifiable risk factors for low birth weight among mothers of different age groups at pregnancy. The number on the top of each column represents the sum of PAF for each risk factor. PAF: population attributable factor

We further explored the contribution of modifiable risk factors for low birth weight, as demonstrated in Fig. 4 and Table 5. The *PAF* estimation indicated that 40.92% of low birth weight cases could be prevented through adherence to healthy lifestyle behaviors and ensuring optimal periconceptional nutrition. Notably, compared to women with advanced gestational age, mothers aged under 35 years presented a stronger joint preventive effect (44.61% vs. 31.91%). In the total population, the largest *PAFs* for low birth weight were associated with weight gain < 10 kg during pregnancy (20.59%, 18.68%−22.45%) and being underweight before conception (7.01%, 5.55%−8.45%). Additionally, low birth weight attributed to pregestational drinking, drinking during the first trimester, imbalanced diet, moderate-to-high physical activity, lack of folic acid supplementation and renovation at home were estimated to be 1.36%, 0.43%, 3.80%, 4.02%, 2.66%, and 1.05%, respectively.

**Table 5:** PAFs of modifiable risk factors for low birth weight among mothers of different age groups at pregnancy

Age group	Modifiable risk factor	*PAF* (95%*CI*)
**Overall**		
	Pregestational drinking	1.36 (0.76–1.95)
	Underweight before conception	7.01 (5.55–8.45)
	Lack of folic acid supplementation	2.66 (1.78–3.53)
	Drinking during the first trimester	0.43 (−0.04–0.91)
	Moderate-to-high physical activity	4.02 (2.36–5.66)
	Weight gain < 10 kg during pregnancy	20.59 (18.68–22.45)
	Imbalanced diet	3.80 (2.48–5.10)
	Renovation at home	1.05 (0.26–1.82)
** < 35 years at pregnancy**		
	Pregestational drinking	1.28 (0.54–2.02)
	Underweight before conception	9.48 (7.53–11.38)
	Lack of folic acid supplementation	2.85 (1.79–3.90)
	Drinking during the first trimester	0.60 (0.02–1.18)
	Moderate-to-high physical activity	4.15 (2.17–6.08)
	Weight gain < 10 kg during pregnancy	21.14 (18.92–23.30)
	Imbalanced diet	4.31 (2.69–5.90)
	Renovation at home	0.80 (−0.14–1.74)
** ≥ 35 years at pregnancy**		
	Pregestational drinking	1.39 (0.39–2.37)
	Underweight before conception	1.33 (−0.35–2.99)
	Lack of folic acid supplementation	1.93 (0.37–3.48)
	Drinking during the first trimester	0.14 (−0.66–0.92)
	Moderate-to-high physical activity	4.20 (1.20–7.11)
	Weight gain < 10 kg during pregnancy	19.01 (15.33–22.53)
	Imbalanced diet	2.25 (0.01–4.45)
	Renovation at home	1.66 (0.23–3.07)

*CI* Confidence interval, *PAF* Population attributable fraction

## Discussion

The present cohort study provides findings with the largest Chinese population thus far, elucidating the maternal pregestational and periconceptional factors associated with low birth weight. Owing to the prospective study design, the results offer relatively convincing evidence of causal inference. Among 34,104 enrolled participants, 3,020 pregnancies (incidence: 8.9%) were affected by low birth weight. The incidence reported in our study was higher than the 5.15% reported in a case–control study [6] and the 4.26% reported in a birth cohort in southern China [11]. This may be due to our limited study site in one maternal and child health care hospital located in Central China.

### Pregestational factors

Maternal pregestational factors were observed to be associated with an increased risk of low birth weight, including a lower education level, urban residence, advanced maternal age at pregnancy (≥ 35 years), multiparity, history of preterm birth, and medical history. Growing evidence have shown that demographic characteristics are major contributors to low birth weight [11]. In contrast to previous research conducted in high-income (Australia) and low-income (Ethiopia) countries documenting living in rural areas as a risk factor [12, 13], urban residence was discovered to increase susceptibility to low birth weight in the Chinese context, according to our findings. We observed that individuals residing in urban areas exhibited a higher prevalence of abnormal BMI before conception compared to those in rural areas (underweight: 14.59% vs. 14.16%, *P* < 0.05; overweight and obesity: 15.39% vs. 15.16%, *P* < 0.05). Furthermore, our study indicated that mothers with abnormal pregestational BMI were at an increased risk of having low birth weight offspring, which may partly account for the association between maternal urban residence and low birth weight. Additionally, air pollution in urban areas may also contribute to this association [29]. In line with prior studies [30], we identified advanced gestational age and multiparity as determinants of low birth weight. In addition, the impact of adverse obstetric history was uncovered in this article. We reported 117% and 56% increased risk for full-term low birth weight births in mothers with a prior history of preterm birth or neonatal death, respectively.

Moreover, we investigated the associations of low birth weight with various pregestational diseases, including hypertensive disorders, diabetes mellitus, kidney disease, hematological disease, antiphospholipid syndrome (APL), tuberculosis, and syphilis. To our knowledge, this is the first study taking into account multisystem diseases. According to birth cohort studies conducted in the US and the UK, maternal pregestational hypertension is correlated with adverse birth outcomes (especially small-for-gestational-age), potentially moderated by weight gain and preeclampsia [31, 32]. We further provide evidence regarding the impact of maternal chronic hypertension on birth weight in Chinese populations. Our results also indicated significant associations of bacterial infections such as tuberculosis and syphilis with low birth weight, in line with previous research [33]. Our findings provide a basis for identifying at-risk pregnant women on the basis of pregestational factors.

### Periconceptional factors

The associations between periconceptional factors and low birth weight were found in this study, encompassing maternal infection, gestational complications, a lack of folic acid supplementation, being underweight before pregnancy, insufficient weight gain and an imbalanced diet during gestation, alcohol consumption, and environmental exposure (i.e., renovation at home). The present study found that maternal infection during early pregnancy was linked to low birth weight. Similarly, prior findings corroborated the finding that reproductive tract and urinary tract infections are associated with adverse birth outcomes [34–36]. In accordance with published research [13, 18], our results showed that gestational complications were risk factors for low birth weight, with the strongest association observed for preeclampsia (*RR* = 5.97). Impaired placental implantation due to preeclampsia disturbs perfusion, resulting in placental ischemia, fetal hypoxia and abnormal intrauterine growth, and subsequently restricted growth at birth and in later life [37, 38]. A previous meta-analysis showed that hyperthyroidism and hypothyroidism during gestation increase the risk of low birth weight, with FT4 and TSH levels inversely correlated with birth weight [19]. Our study, however, reached the opposite conclusions, as 38% and 15% declined incidence was found for gestational hyperthyroidism and hypothyroidism. Another interesting yet suspicious finding in this research was a decrease of 47% in low birth weight among mothers with placenta previa, which is commonly regarded to induce adverse birth outcomes [39]. These differences are likely attributable to the underlying moderating or mediating effects of other factors, expecting future research.

Our results indicated that markers of unfavorable periconceptional nutritional status, including pregestational underweight, a lack of folic acid supplementation, an imbalanced diet and insufficient weight gain during pregnancy, significantly contributed to low birth weight, which is in accordance with the findings of previous studies [15, 16, 40, 41]. This may be attributable to the fact that maternal diet and nutritional status during pregnancy can influence infant outcomes by modulating the diversity of gut microbiota [42]. Inadequate nutrition during pregnancy may be related to lower socioeconomic status, as well as selective nutritional regimens such as vegetarian lifestyles [43]. Furthermore, we found a plausible dose–effect relationship, as overweight and obese mothers were 13% and 49% less risky to have low birth weight offspring (*P*_for_trend < 0.001), which was different from the U-shaped trend observed in previous articles [44, 45]. The present study highlights the necessity of sufficient periconceptional nutrition and regular folic acid supplementation to optimize birth weight in offspring.

To the best of our knowledge, this study provides the initial association between periconceptional medication and low birth weight. The use of oral contraceptives, ovulation stimulants, and macrolides antibiotics before conception was associated with low birth weight in univariate analyses but did not reach statistical significance in multivariate models. This suggests that women planning pregnancy should avoid taking these medications to reduce the occurrence of low birth weight. Our study also examined the possible hazard of exposure to environmental pollutants and found that household renovation raised the odds of low birth weight by 21%, similar to previous research [46, 47]. We expect further analysis incorporating measurable internal exposure to household pollutants during early gestation and proposing safe cutoff values for pregnant women.

### Lifestyle factors

Our study examined the associations of low birth weight with modifiable lifestyle behaviors and reported that consuming alcohol before and during pregnancy and moderate-to-high physical activity during the first trimester increased the risk by 54%, 23%, and 22%, respectively. Although publications abroad have confirmed the role of maternal smoking in low birth weight [20, 48], such a correlation was not statistically significant herein, potentially because of the minimal proportion of smoking women in China. Drinking alcohol (defined as ≥ 2 g per day) before pregnancy was a determinant of low birth weight in our research, which differs from the findings of a study conducted in Norway showing the protective impact of pregestational drinking [49], and confounding effects of socioeconomic factors might be accountable. Moreover, a Spanish survey demonstrated that pregnant women with alcohol consumption < 6 g/day presented a reduced risk, whereas those with consumption ≥ 12 g/day exhibited 1.67-fold greater risk for low birth weight [50], revealing the drastic distinctions between populations and the importance of research exclusively on Chinese women. This study has also pointed out the hazard of excessive exercise during early gestation, providing epidemiological evidence for health education.

### Comparison between preterm and non-preterm births

Overall, determinants of low birth weight among preterm and full-term births show different patterns. Several factors, comprising pregestational lifestyle behaviors such as tobacco and alcohol use, and infections during the first trimester were significantly associated with low birth weight only among preterm neonates, suggesting the potential mediating role of preterm birth. Besides, modifiable factors including a lack of folic acid supplementation, moderate-to-high physical activity during pregnancy, and household renovation were uncovered to contribute to low birth weight among full-term infants. Additionally, mothers aged over 35 years at pregnancy were more likely to deliver preterm births with low birth weight, underscoring that particular attention should be given to women of advanced maternal age.

### Public health enlightenment

The evaluation on *PAFs* of modifiable risk factors indicated that insufficient weight gain during pregnancy accounts for 20.59% of cases with low birth weight, highlighting the significance of adequate nutrition during pregnancy and regular nutritional screening. Furthermore, to optimize the effects of interventions, women aged under 35 years should aim for an ideal BMI when preparing for pregnancy, while those of advanced maternal age should focus on their periconceptional diet. Moreover, moderate-to-high physical activity should be avoided during the first trimester, irrespective of gestational age.

### Limitations

Despite the strengths of the prospective design, this study carries the following limitations. Firstly, the present study was conducted in one provincial maternal and child health care hospital in Central China, which means the findings of this study lack nationwide representativeness, thereby limiting the generalizability of the conclusions. Secondly, since participants were recruited upon their initial visits to prenatal care, information on pregestational factors, including weight and height (for calculating BMI), medical history and lifestyle behaviors, was inevitably affected by recall bias. In addition, our results might be affected by self-report bias. Thirdly, the investigation of dietary factors is typically conducted using 24-h dietary recalls or dietary records. However, in this study, we did not collect quantitative data on food consumption due to the large sample size (initially comprising 49,158 participants) and limited resources. Future research should focus on more detailed surveys to explore the associations between maternal intake of specific nutrients and low birth weight, informing targeted nutritional interventions. Finally, factors associated with very/extremely low birth weight (altogether 645 newborns) were not further analyzed in light of the inadequate sample size.

## Conclusions

In conclusion, maternal advanced gestational age, lower education, urban residence, adverse obstetric history, pregestational and periconceptional diseases, insufficient gestational nutrition, and unhealthy lifestyle behaviors were associated with an increased risk of low birth weight in offspring. This could be prevented through nutritional and lifestyle interventions during pregnancy, including achieving adequate weight gain, abstaining from tobacco and alcohol consumption, and avoiding excessive physical activity. Our research provides epidemiological evidence to inform the development of targeted interventions aimed at reducing the incidence of low birth weight.

## Supplementary Information

Additional file 1: Supplementary Table 1. Collinearity diagnosis of variables within each dimension using *K-M-O* test and Bartlett test.

## Data Availability

Data are unavailable due to privacy or ethical restrictions.

## Abbreviations

BMI: Body mass index
CI: Confidence interval
PAF: Population attributable fraction
RR: Relative risk
