# Supplementary material for: The impact of maternal health and lifestyle on low birth weight: a prospective cohort study
Source: Ital J Pediatr. 2025 Jul 10;51:217. doi: 10.1186/s13052-025-02080-x (PMC12247386; doi:10.1186/s13052-025-02080-x)
Supplement: Supplementary file 1 — Additional file 1: Supplementary Table 1. Collinearity diagnosis of variables within each dimension using K-M-O test and Bartlett test. [file 13052_2025_2080_MOESM1_ESM.docx]

**Additional file 1**

**Supplementary Table 1** Collinearity diagnosis of variables within each dimension using *K-M-O* test and Bartlett test

| **Dimension** | **Variables** | ***K-M-O* statistic** | **Bartlett test** |
| --- | --- | --- | --- |
| **All** | All variables | 0.540 | <.001 |
| **Constitutional factors** |  |  |  |
|  | Age at pregnancy | 0.503 | <.001 |
|  | Education degree |  |  |
|  | Residence |  |  |
| **History of gestational complications** |  |  |  |
|  | History of gestational diabetes mellitus | 0.488 | <.001 |
|  | History of gestational hypertension |  |  |
|  | History of placenta previa |  |  |
|  | History of placental abruption |  |  |
|  | History of premature rupture of membrane |  |  |
|  | History of gestational anemia |  |  |
|  | History of antepartum or postpartum hemorrhage |  |  |
|  | History of abortion |  |  |
|  | History of ectopic pregnancy |  |  |
|  | History of intrauterine growth restriction |  |  |
| **History of adverse pregnancy outcomes** |  |  |  |
|  | History of preterm birth | 0.500 | <.001 |
|  | History of low birth weight |  |  |
|  | History of neonatal death |  |  |
| **Pregestational diseases** |  |  |  |
|  | Hypertension | 0.499 | <.001 |
|  | Heart disease |  |  |
|  | Kidney disease |  |  |
|  | Hematological disease |  |  |
|  | Systemic lupus erythematosus, SLE |  |  |
|  | Antiphospholipid syndrome, APL |  |  |
|  | Thyroid disease |  |  |
|  | Tuberculosis |  |  |
|  | Syphilis |  |  |
| **Pregestational lifestyle** |  |  |  |
|  | Smoking 3 months before pregnancy | 0.499 | <.001 |
|  | Passive smoking 3 months before pregnancy |  |  |
|  | Drinking 3 months before pregnancy |  |  |
| **Diseases during the first trimester** |  |  |  |
|  | Systematic infection | 0.497 | <.001 |
|  | Respiratory infection |  |  |
|  | Urinary tract infection |  |  |
|  | Reproductive tract infection |  |  |
|  | Coxsackievirus-B infection |  |  |
| **Gestational complications** |  |  |  |
|  | Gestational anemia | 0.490 | <.001 |
|  | Preeclampsia |  |  |
|  | Gestational hyperthyroidism |  |  |
|  | Gestational hypothyroidism |  |  |
|  | Intrahepatic cholestasis of pregnancy |  |  |
|  | Venous thromboembolism of pregnancy |  |  |
|  | Placenta previa |  |  |
|  | Placental abruption |  |  |
|  | Premature rupture of membrane |  |  |
| **Periconceptional medication** |  |  |  |
|  | Lack of folic acid supplementation | 0.497 | <.001 |
|  | Oral contraceptives intake 3 months before conception |  |  |
|  | Ovulation stimulants intake 3 months before conception |  |  |
|  | Macrolides antibiotics intake 3 months before conception |  |  |
| **Periconceptional nutrition** |  |  |  |
|  | BMI before conception | 0.502 | <.001 |
|  | Weight gain during pregnancy |  |  |
|  | Imbalanced diet |  |  |
| **Periconceptional lifestyle** |  |  |  |
|  | Drinking during the first trimester | 0.500 | 0.001 |
|  | Physical activity during the first trimester |  |  |
| **Periconceptional exposure to environmental hazards** | Renovation at home | NA | NA |
